# Supplementary material for: Unilateral hamstring foam rolling does not impair strength but the rate of force development of the contralateral muscle
Source: PeerJ. 2019 May 29;7:e7028. doi: 10.7717/peerj.7028 (PMC6545114; doi:10.7717/peerj.7028)
Supplement: Supplemental Information 1 — Pre-exercise Health Status Questionnaire. [file peerj-07-7028-s007.doc]

#

**PRE-EXERCISE TESTING HEALTH & EXERCISE STATUS QUESTIONNAIRE**

**The University of Mississippi**

**Department of Health, Exercise Science & Recreation Management**

# Neuromuscular Laboratory

Name ________________________________________________ Date______________

Home Address __________________________________________________________________

Phone _______________________

Person to contact in case of emergency __________________________________________

Emergency Contact Phone ______________________ Birthday (mm/dd/yy)____/_____/_____

Gender ________ Age ______(yrs) Height ______(ft)______(in) Weight______(lbs)

Does the above weight indicate: a gain____ a loss____ no change____ in the past year?

If a change, how many pounds?___________(lbs)

**A. JOINT-MUSCLE STATUS** (**🗸**Check areas where you currently have problems)

Joint Areas Muscle Areas

( ) Wrists ( ) Arms

( ) Elbows ( ) Shoulders

( ) Shoulders ( ) Chest

( ) Upper Spine & Neck ( ) Upper Back & Neck

( ) Lower Spine ( ) Abdominal Regions

( ) Hips ( ) Lower Back

( ) Knees ( ) Buttocks

( ) Ankles ( ) Thighs

( ) Feet ( ) Lower Leg

( ) Other_______________________ ( ) Feet

( ) Other_____________________

**B. HEALTH STATUS** (**🗸**Check if you currently have any of the following conditions)

( ) High Blood Pressure ( ) Acute Infection

( ) Heart Disease or Dysfunction ( ) Diabetes or Blood Sugar Level Abnormality

( ) Peripheral Circulatory Disorder ( ) Anemia

( ) Lung Disease or Dysfunction ( ) Hernias

( ) Arthritis or Gout ( ) Thyroid Dysfunction

( ) Edema ( ) Pancreas Dysfunction

( ) Epilepsy ( ) Liver Dysfunction

( ) Multiply Sclerosis ( ) Kidney Dysfunction

( ) High Blood Cholesterol or ( ) Phenylketonuria (PKU)

Triglyceride Levels ( ) Loss of Consciousness

( ) Allergic reactions to rubbing alcohol

* *NOTE: If any of these conditions are checked, then a physician’s health clearance will be required.*

# C. PHYSICAL EXAMINATION HISTORY

Approximate date of your last physical examination______________________________

Physical problems noted at that time__________________________________________

Has a physician ever made any recommendations relative to limiting your level of physical exertion? _________YES __________NO

If YES, what limitations were recommended?___________________________________

________________________________________________________________________

**D. CURRENT MEDICATION USAGE** (List the drug name and the condition being managed)

## MEDICATION CONDITION

__________________________ ____________________________________

__________________________ ____________________________________

__________________________ ____________________________________

**E. PHYSICAL PERCEPTIONS** (Indicate any unusual sensations or perceptions. **🗸**Check if you have recently experienced any of the following during or soon after *physical activity* (PA); or during *sedentary periods* (SED))

PA SED PA SED

( ) ( ) Chest Pain ( ) ( ) Nausea

( ) ( ) Heart Palpitations ( ) ( ) Light Headedness

( ) ( ) Unusually Rapid Breathing ( ) ( ) Loss of Consciousness

( ) ( ) Overheating ( ) ( ) Loss of Balance

( ) ( ) Muscle Cramping ( ) ( ) Loss of Coordination

( ) ( ) Muscle Pain ( ) ( ) Extreme Weakness

( ) ( ) Joint Pain ( ) ( ) Numbness

( ) ( ) Other________________________ ( ) ( ) Mental Confusion

**F. EXERCISE STATUS**

**Do you regularly engage in aerobic forms of exercise (i.e., jogging, cycling, walking, etc.)? YES NO**

How long have you engaged in this form of exercise? ______ years ______ months

How many hours per week do you spend for this type of exercise? _______ hours

**Do you regularly lift weights? YES NO**

How long have you engaged in this form of exercise? ______ years ______ months

How many hours per week do you spend for this type of exercise? _______ hours

**Do you regularly play recreational sports (i.e., basketball, racquetball, volleyball, etc.)? YES NO**

How long have you engaged in this form of exercise? ______ years ______ months

How many hours per week do you spend for this type of exercise? _______ hours

**Do you regularly perform any types of stretching exercise (i.e., yoga, pre-exercise warmup, post-exercise stretch)? YES NO**

How long have you engaged in this form of exercise? ______ years ______ months

How many hours per week do you spend for this type of exercise? _______ hours ______ minutes
